# Cross-Modal Image Fusion via Structure Preservation and Detail Enhancement Optimization

**DOI:** 10.3390/s26154880

**Published:** 2026-08-03

**Authors:** Xiaoxia Wang, Shuang Guo, Fengbao Yang, Bo Li, Yuhui Yan

**Affiliations:** School of Information and Communication Engineering, North University of China, Taiyuan 030051, China; sz202405019@st.nuc.edu.cn (S.G.); yfengb@163.com (F.Y.); libo_5y@nuc.edu.cn (B.L.); sz202405082@st.nuc.edu.cn (Y.Y.)

**Keywords:** infrared and visible image fusion, wavelet transform, Mamba, rectified flow

## Abstract

**Highlights:**

**What are the main findings?**

- Proposes Structure–Detail Constrained Fusion (SDC-Fusion), a frequency-decoupled infrared and visible image fusion framework that separately models low-frequency structure and high-frequency detail.
- Restricts Rectified Flow to the high-frequency wavelet subbands and learns a gated few-step residual compensation trajectory from the visible high-frequency component to a local-directional-energy-guided target, rather than generating the complete fused image or latent representation.
- Develops a low-frequency structure preservation module that integrates a four-directional Mamba scan with multi-dilated depthwise convolutions, simultaneously maintaining global luminance integrity and optimizing local grayscale transitions.
- Ranks first on five of seven metrics and second on the remaining two metrics on both the MSRS and M^3^FD datasets, while using 0.535 M parameters and 67.5 G FLOPs.

**Abstract:**

Existing end-to-end infrared–visible fusion methods often blur edges, smooth textures and weaken target-to-background contrast. We therefore propose Structure–Detail Constrained Fusion (SDC-Fusion), a frequency-decoupled framework with separate constraints on structure and detail. The proposed method employs the Haar wavelet transform to decompose the source images into low-frequency structural and high-frequency detail components. The high-frequency branch uses a local directional-energy prior to construct the target guidance. Unlike existing Rectified Flow-based approaches that operate on the full image or a generic latent representation, our gated module applies Rectified Flow only to the high-frequency wavelet subbands. It learns a few-step residual trajectory from the visible high-frequency coefficients to the target representation, enhancing infrared target boundaries and visible textures without altering low-frequency structure. In the low-frequency branch, adaptive weighting, four-directional Mamba scanning, and multi-dilation depthwise convolutions are integrated to preserve global luminance and background structure while optimizing local grayscale transitions. Comparative experiments against eleven representative fusion methods on the MSRS and M^3^FD datasets show that SDC-Fusion ranks first in SSIM, VIF, Q_abf_, SF, and PSNR on MSRS, and first in SSIM, VIF, Q_abf_, SD, and PSNR on M^3^FD, while ranking second in the remaining two metrics on each dataset. Relative to the strongest competing result, the largest improvements reach 10.44% in SF on MSRS and 5.72% in VIF on M^3^FD. The model contains 0.535 M parameters and requires 67.5 G FLOPs.

## 1. Introduction

Infrared and visible image fusion represents a critical task in the field of multimodal intelligent perception. It aims to integrate thermal radiation targets from infrared imaging with textures, edges, and background structures from visible imaging into a single unified image. Because infrared imaging is less dependent on visible illumination, it can reliably reveal thermal targets such as pedestrians and vehicles at night and under low-light or adverse-weather conditions [1]. Conversely, visible images provide higher spatial resolution and texture representations that align closely with human visual perception, offering detailed road contours, building edges, and complex background scenes. Infrared–visible fusion has therefore been investigated for applications including nighttime road perception, surveillance, reconnaissance, unmanned-aerial-vehicle inspection and downstream object detection [2,3,4,5,6]. However, infrared and visible images exhibit significant differences in terms of imaging mechanisms and frequency distributions. Thermal targets in infrared images and complex textures in visible images frequently interfere with each other during the fusion process. Thus, simultaneously emphasizing salient thermal targets and preserving fine texture and structural consistency remains a central challenge in infrared–visible image fusion [7].

**Figure 1:**
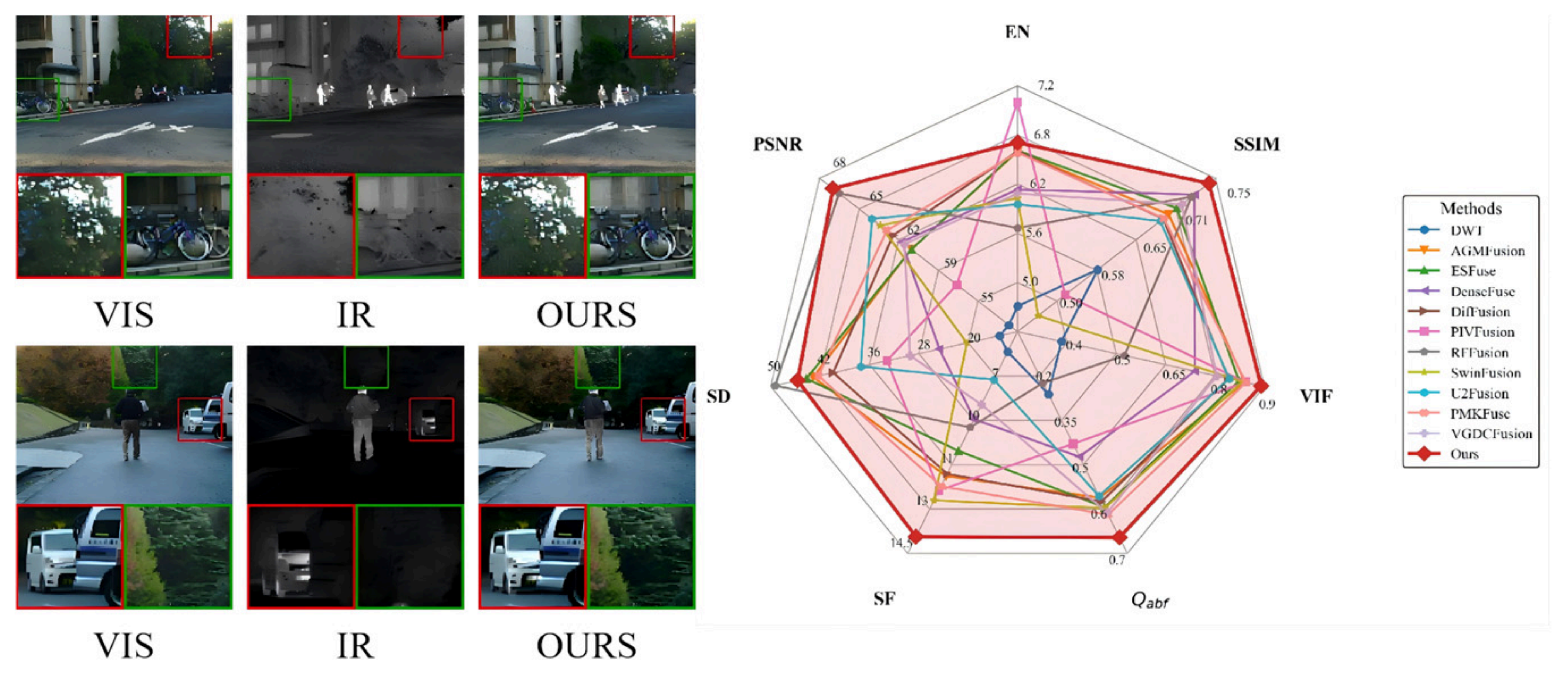
Representative fusion results and quantitative comparisons on two scenes from the MSRS test set. The (**left**) panels show the visible images, infrared images, and fusion results generated by SDC-Fusion, while the (**right**) panels present radar-chart comparisons with competing methods across seven distinct metrics.

Existing infrared and visible image fusion methods primarily comprise traditional multi-scale transform approaches and deep learning methods [8]. For traditional methods, the fusion rules are typically fixed during the algorithm design phase, making it difficult to adapt to varying texture intensities and shifting thermal target scales across complex scenarios. Meanwhile, deep learning frameworks still tend to process both source modalities within a unified pixel or feature domain, leading to the undifferentiated blending of infrared thermal radiation responses, visible texture backgrounds, and localized noise. Processing both modalities in a common pixel or feature space can induce competition between them: salient infrared responses may obscure visible textures, whereas complex visible backgrounds may suppress thermal targets. Noise amplification during feature fusion can further reduce target-to-background contrast, producing blurred edges and smoothed details [9,10,11]. Figure 1 presents the fusion results of two sample images generated by SDC-Fusion, alongside a quantitative comparison with eleven competing methods.

To address these limitations, we propose a frequency-decoupled fusion framework with separate constraints on structure and detail. A Haar wavelet transform decomposes the infrared image and visible luminance channel into low-frequency structural components and high-frequency detail components, which are then processed independently. Starting from the visible high-frequency component, the high-frequency branch incorporates a gated Rectified Flow module to learn a few-step residual mapping from visible textures to the target high-frequency representation. Concurrently, the low-frequency branch utilizes a Mamba state-space model coupled with local multi-dilation depthwise convolutions to stabilize target-to-background contrast while preserving the global structure and background luminance hierarchy. Consequently, the proposed method enhances infrared target edges and visible texture details while maintaining the low-frequency response of target regions and the hierarchical background luminance. The primary contributions of this study are summarized as follows:

- We introduce a frequency-decoupled fusion framework that combines high-frequency residual compensation with low-frequency global–local structure preservation, reducing edge blur, texture smoothing and contrast loss.
- We develop a gated Rectified Flow module for high-frequency residual compensation. A local directional-energy prior defines the target endpoint, and the gated velocity field controls a few-step residual trajectory that sharpens edges and fine textures. Restricting the flow to high-frequency wavelet subbands prevents the updates from altering low-frequency luminance and large-scale structure.
- A low-frequency structure preservation module based on Mamba and multi-dilated convolutions is proposed. Building upon an adaptive weight fusion strategy, this module leverages a four-directional Mamba global scan alongside multi-dilated depthwise convolutions to execute residual corrections on the initial fused features, thereby simultaneously preserving global structural integrity and optimizing local grayscale transitions.
- Comprehensive comparative experiments are conducted on the MSRS and M^3^FD datasets against eleven representative fusion methods. On MSRS, SDC-Fusion ranks first in SSIM, VIF, Q_abf_, SF, and PSNR and second in EN and SD. On M^3^FD, it ranks first in SSIM, VIF, Q_abf_, SD, and PSNR and second in EN and SF. The proposed model contains 0.535 M parameters and requires 67.5 G FLOPs, demonstrating a favorable balance between fusion performance and computational complexity.

## 2. Related Work

### 2.1. Traditional IVIF Methods

Early infrared and visible image fusion predominantly relied on hand-crafted image representations and fusion rules. Traditional methodologies generally encompass spatial-domain weighting, transform-domain decomposition [12], sparse representation, and saliency detection. The primary advantages of these methods lie in their explicit workflows and high interpretability, as well as their independence from large-scale training datasets. However, manual fusion rules are typically fixed during the algorithm design phase, severely restricting their capacity to adapt to scale variations in thermal targets, the degradation of visible textures, and the accumulation of sensor noise in complex environments. Consequently, although traditional frameworks provide valuable insights into frequency decomposition and structural priors, relying exclusively on static rules fails to satisfy the demand for stable detail preservation in complex scenarios [13,14].

### 2.2. Transformer-Based IVIF Methods

Benefiting from robust feature extraction capabilities, deep learning has become the dominant paradigm in contemporary image fusion. For instance, Wang et al. [15] introduced SwinFuse, which utilizes residual Transformer modules to extract global features. Similarly, Ma et al. [16] proposed SwinFusion, establishing a long-range cross-learning framework that integrates self-attention and cross-attention to combine the local characteristics of convolutional neural networks with the global representations of Swin Transformers. Additionally, Hua et al. [17] proposed PIVFusion, which jointly optimizes low-light enhancement and image fusion through perceptual transformation. Although Transformers enhance global interactions via self-attention mechanisms, their computational complexity grows significantly with increasing spatial resolution [18,19,20,21,22,23].

### 2.3. Mamba-Based IVIF Methods

Structured state-space models (SSMs) have recently been adopted for image fusion because they model long-range dependencies with linear rather than quadratic complexity. For instance, Peng et al. [24] proposed FusionMamba to achieve efficient feature interaction with linear state-space modeling. Furthermore, Li et al. [25] proposed MambaDFuse, establishing a specialized dual-phase Mamba fusion model to separately process distinct feature spaces. Concurrently, these methods model long-range dependencies through a selective state update mechanism, thereby capturing backgrounds and large-scale structural configurations more coherently [26].

### 2.4. Generative Fusion-Based IVIF Methods

Generative adversarial network-based image fusion methods leverage adversarial learning principles to generate fused images through adversarial training between a generator and a discriminator [27]. Ma et al. [28] pioneered this direction by proposing FusionGAN, which employed a generator to synthesize the fused image and a discriminator to evaluate whether the distribution of the fused result aligned with that of the source images. However, approaches based on generative adversarial networks often suffer from a lack of interpretability and unstable training dynamics, subsequently degrading the quality of image fusion.

Ho et al. [29] introduced denoising diffusion probabilistic models (DDPM), which generate images by applying a sequence of denoising steps to a noisy initial state. Diffusion models possess an interpretable training mechanism while offering superior stability. Building on this framework, Yue et al. [30] proposed DifFusion, introducing multi-channel data into the diffusion framework to achieve high-fidelity image fusion. Similarly, Zhao et al. [31] proposed DDFM, designing an unconditional generation module alongside a conditional likelihood correction module. Nevertheless, diffusion models entail high computational overhead in terms of storage requirements and inference time.

Rectified flow and flow matching provide a more direct modeling approach for generative fusion by learning velocity fields between samples to establish trajectories from the starting distribution to the target distribution. Wang et al. [32] introduced RFFusion, integrating rectified flow into image fusion tasks and demonstrating that straightening the generation trajectory reduces the redundant sampling overhead inherent in diffusion-based fusion. Notably, existing generative fusion paradigms predominantly model either the entire image or a generic latent space [33]. In infrared and visible image fusion, source modalities already provide most of the structural layout of the scene, implying that regions requiring compensation are primarily concentrated in high-frequency components, such as edges, textures, and object contours.

## 3. Method

### 3.1. Overall Framework

Infrared and visible image fusion aims to construct a fused image $I_{f}$ that simultaneously preserves the thermal target responses of the infrared modality and the textural details of the visible modality. Given a pair of co-registered source images, comprising the infrared image $I_{\mathrm{ir}}\in R^{1\times H\times W}$ and the visible luminance image $I_{\mathrm{vis}}\in R^{1\times H\times W}$, this process can be formulated as follows:

(1) $$\;I_{f\;}=\;F_{\Theta }\left(I_{ir},I_{vis}\right)$$

**Figure 2:**
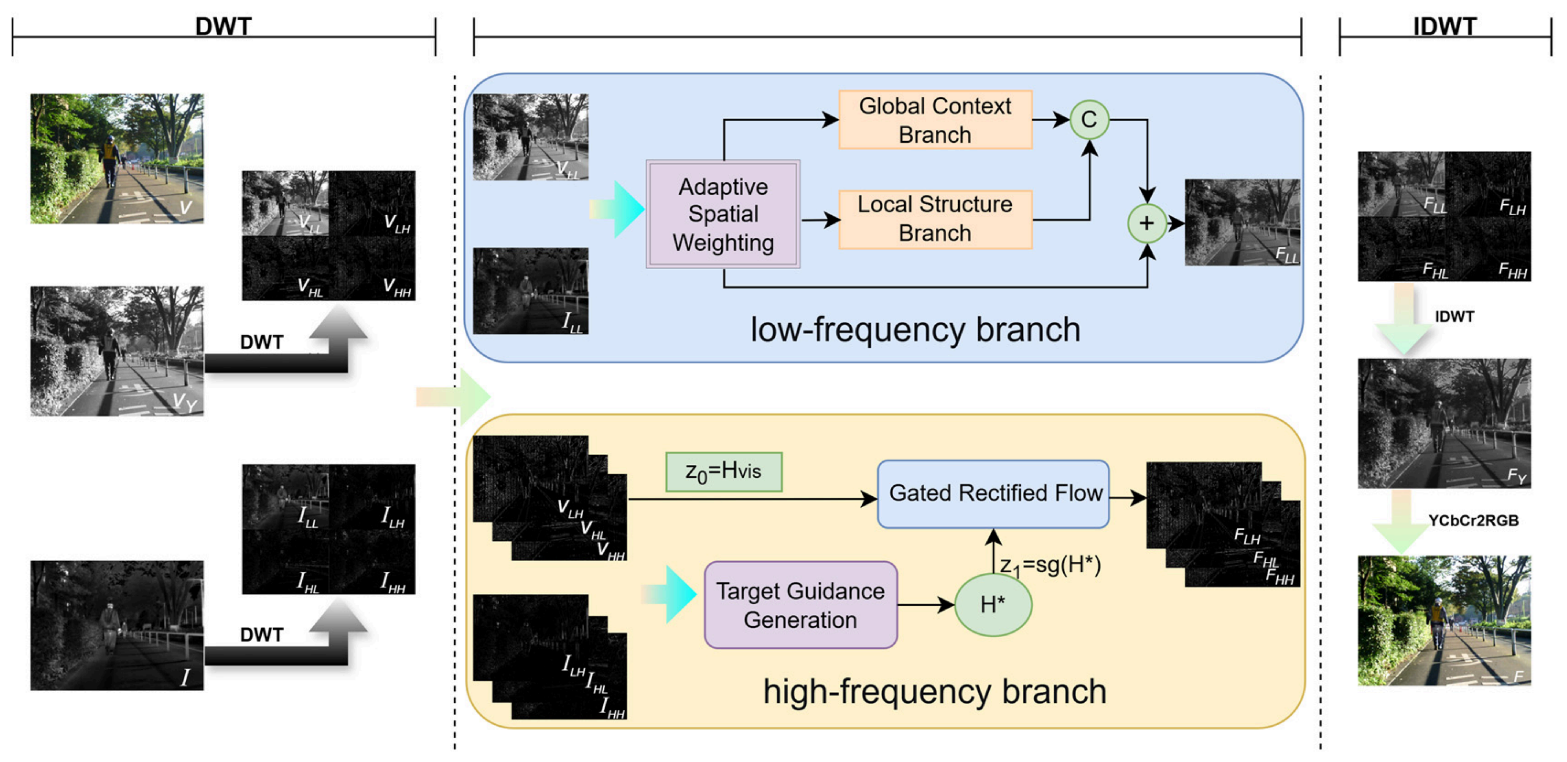
Schematic illustration of the overall framework of the proposed SDC-Fusion method.

This formulation establishes the overall learning objective of the proposed method. For color inputs, only the luminance channel is fused. The fused luminance is then recombined with the original chrominance channels to produce the final color image [34,35]. The comprehensive workflow of the proposed method is illustrated in Figure 2.

The framework decomposes the source images into low-frequency structural and high-frequency detailed components via the Haar wavelet transform. The fused low- and high-frequency components are subsequently reconstructed via the inverse wavelet transform to generate the final fused image. The overall data flow is summarized as follows:

(2) $$\left(L_{ir},H_{ir}\right)\;=\;D\left(I_{ir}\right),\;\left(L_{vis},H_{vis}\right)\;=\;D\left(I_{vis}\right)$$

(3) $$\;L_{f}=M_{L}\left(L_{ir},L_{vis}\right)$$

(4) $$H_{f}=M_{H}\left(H_{ir},H_{vis}\right)$$

(5) $$\;I_{f}=D^{-1}\left(L_{f},H_{f}\right)$$

This formulation outlines the primary computational pipeline of the proposed framework. Specifically, D and $D^{-1}$ denote the wavelet decomposition and inverse wavelet reconstruction operators, respectively, while $M_{L}$ and $M_{H}$ represent the low-frequency and high-frequency processing branches.

### 3.2. Haar Wavelet Frequency Decomposition

The proposed framework utilizes a first-level Haar wavelet transform to achieve an explicit decomposition of the source images as follows [36]:

(6) $$\;\left(L,H\right)\;=\;D\left(I\right),\;H\;=\;\left[H^{LH},H^{HL},H^{HH}\right]$$

where L denotes the low-frequency subband of LL, which primarily preserves the luminance distribution, background contours, and large-scale structures, while $H^{LH},H^{HL},H^{HH}$ represents the high-frequency subbands across three distinct orientations, predominantly capturing sharp edges and fine textures.

### 3.3. High-Frequency Detail Compensation Module

The high-frequency branch is specifically designed to address the challenges of texture smoothing and detail attenuation in fused images. The proposed framework first constructs a target high-frequency component constrained by the source images, and subsequently learns the high-frequency residual compensation trajectory via a gated Rectified Flow mechanism.

#### 3.3.1. Target High-Frequency Guidance

**Figure 3:**
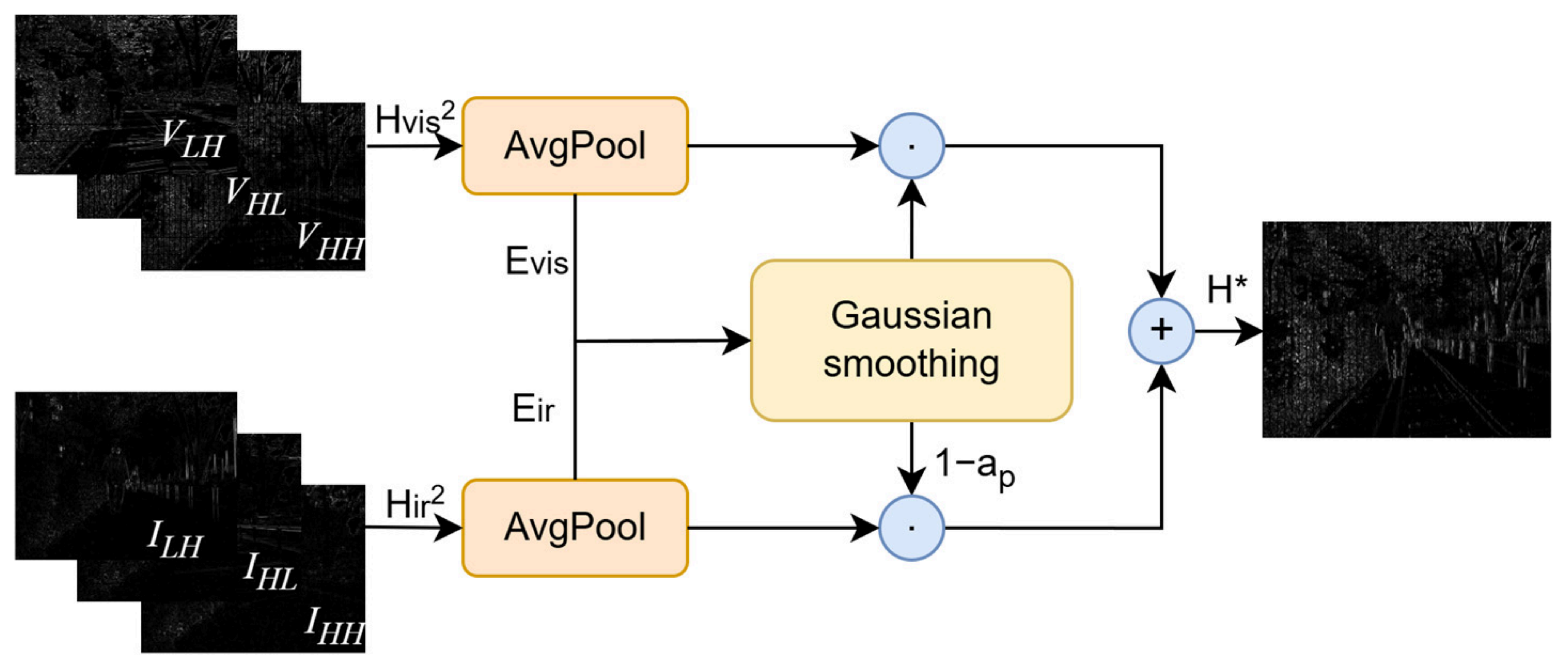
Detailed architecture of the proposed target high-frequency guidance module.

The target high-frequency guidance is designed to provide an explicit endpoint trajectory for the Rectified Flow process. The design of the target high-frequency guidance was informed by the prior-guided reconstruction principle exemplified by FDCNN [37], which employs an interpretable feature-masking layer to guide hierarchical ultrasound-image denoising. In SDC-Fusion, this principle is adapted to infrared–visible image fusion by constructing a local-directional-energy-guided high-frequency target from the source modalities and using it as the endpoint constraint for gated Rectified Flow residual compensation. The generation pipeline of the target high-frequency guidance map is illustrated in Figure 3.

The proposed framework utilizes local directional energy, which is defined as follows:

(7) $$\;E_{ir}={AvgPool}_{3\times 3,s=1}\left({\left|H_{ir}\right|}^{2}\right),\;E_{vis}={AvgPool}_{3\times 3,s=1}\left({\left|H_{vis}\right|}^{2}\right)$$

This formulation quantifies the high-frequency intensity of both infrared and visible modalities at each spatial location and within each directional subband. ${AvgPool}_{3\times 3,s=1}\left(\cdot \right)$ denotes a fixed and non-learnable average-pooling operation with a 3 × 3 kernel and a stride of one. One-pixel reflection padding is applied before pooling to preserve the spatial resolution of the energy maps. The operation is independently performed on the LH, HL, and HH channels without cross-channel interaction. Squaring the high-frequency coefficients converts the signed wavelet responses into nonnegative energy measurements, while local averaging reduces the influence of isolated coefficient fluctuations. On this basis, the initial modality weights are subsequently derived as follows:

(8) $$\;{\tilde{\alpha }}_{p}=\frac{E_{ir}\left(p\right)+\epsilon }{E_{ir}\left(p\right)+E_{vis}\left(p\right)+2\epsilon },\;\alpha_{p}=\left(G_{5,1}*\tilde{\alpha }\right)\left(p\right)$$

where p denotes the spatial position, and ϵ is a small positive constant introduced to prevent numerical instability in the normalization. $G_{5,1}$ denotes a fixed and normalized two-dimensional Gaussian kernel with a spatial size of 5 × 5 and a standard deviation of 1.0. The Gaussian smoothing is independently applied to the three directional weight maps through channel-wise depthwise convolution, without mixing information across the LH, HL, and HH subbands. A two-pixel reflection padding and a stride of one are used to preserve the spatial resolution of the weight maps. The Gaussian kernel is fixed throughout training and is not treated as a learnable network parameter. This local smoothing operation suppresses isolated fluctuations in the energy-ratio maps and improves the spatial continuity of the modality weights.

Consequently, the target high-frequency component is obtained as follows:

(9) $$\begin{array}{c} \;H^{*}=\alpha_{p}⊙H_{ir}+\left(1\;-\;\alpha_{p}\right)⊙H_{vis} \end{array}$$

This formulation establishes a critical constraint for the high-frequency branch. Since $H^{*}$ is inherently constructed by combining the high-frequency components of the source images, the risk of introducing high-frequency artifacts is substantially mitigated. Consequently, the subsequent Rectified Flow process specifically learns the residual trajectory from the visible high-frequency components to this predefined target high-frequency state, instead of reconstructing the entire image from scratch.

#### 3.3.2. Gated High-Frequency Rectified Flow

**Figure 4:**
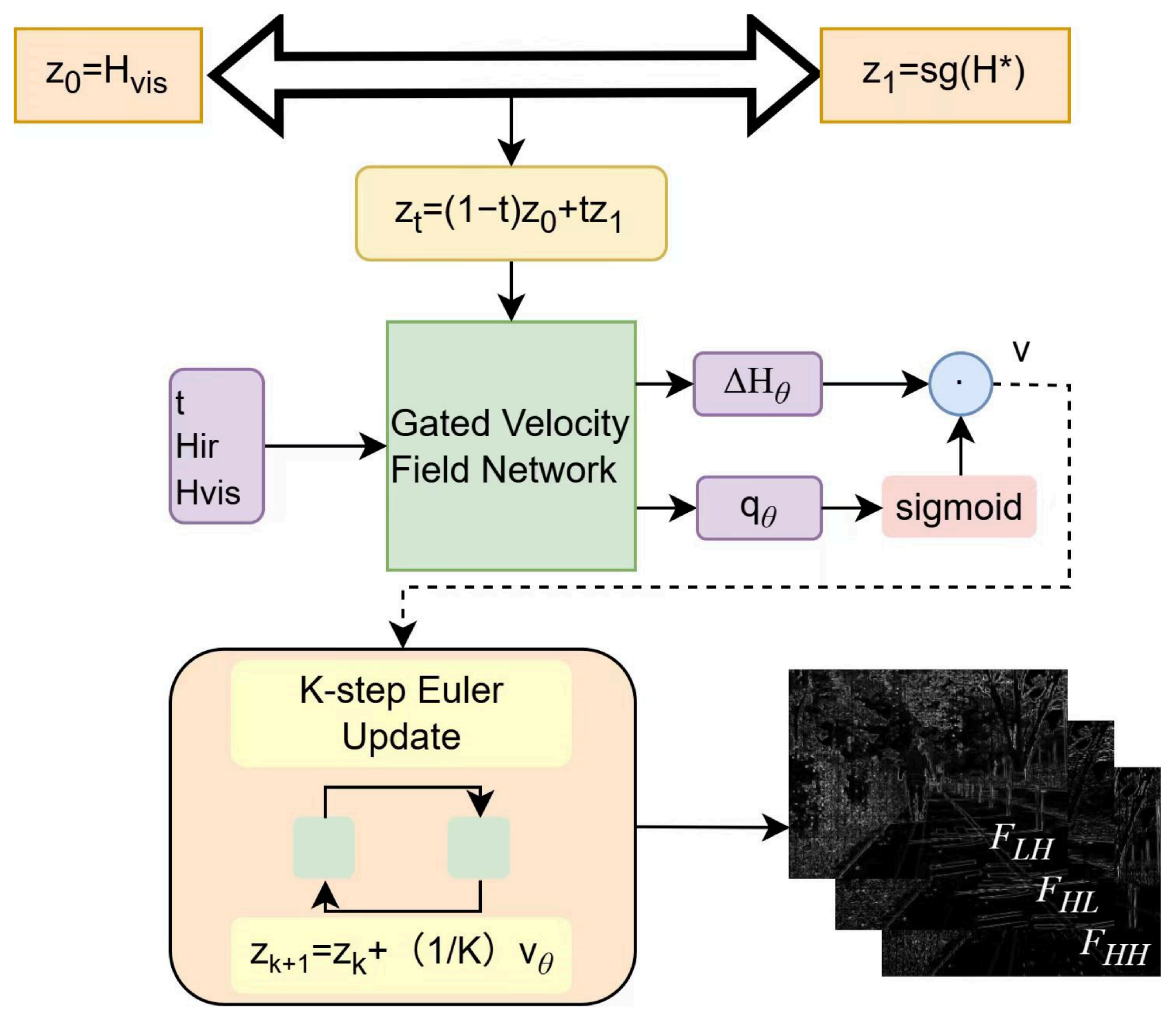
Detailed architecture of the gated high-frequency Rectified Flow module.

To characterize the residual update trajectory from visible textures to target high-frequency components, the proposed framework integrates the Rectified Flow paradigm into the high-frequency domain [38,39,40,41]. The architecture of the gated high-frequency Rectified Flow module is illustrated in Figure 4.

The starting point and the endpoint are defined as follows:

(10) $$\;z_{0}\;=\;H_{vis},\;z_{1}\;=\;\mathrm{sg}\left(H^{*}\right)$$

where $z_{0}$ represents the baseline visible textures, $z_{1}$ denotes the target high-frequency guidance, and sg(∙) signifies the stop-gradient operator employed to stabilize the high-frequency endpoint. During the training phase, a continuous state is constructed between these two endpoints as follows:

(11) $$\;z_{t}\;=\;\left(1-t\right)z_{0}\;+\;tz_{1},\;v^{*}\;=\;z_{1\;}-z_{0}$$

This formulation delineates the learning trajectory and target velocity of Rectified Flow, effectively quantifying the precise high-frequency compensation required to transition from the visible modality to the target state. Conditioned on $z_{t}$, the temporal parameter t, and both infrared and visible high-frequency components, the velocity field network predicts the corresponding residual velocity. Furthermore, a gating mechanism is introduced to modulate the intensity of these updates across distinct spatial regions as follows:

(12) $$\begin{array}{c} \;g_{\theta }=g_{0}+\left(1-g_{0}\right)\sigma \left(q_{\theta }\right),\;v_{\theta }=g_{\theta }⊙\Delta H_{\theta } \end{array}$$

here, $\Delta H_{\theta }$ and $q_{\theta }$ denote the candidate high-frequency residual velocity and the raw gating logit predicted by the velocity-field network, respectively, while $\sigma \left(\cdot \right)$ denotes the sigmoid function. The lower-bound coefficient is fixed as $g_{0}=0.10$ throughout training and inference and is not a learnable parameter. Consequently, the resulting gating values satisfy $g_{\theta }\in (0.1,\;1)$. The nonzero lower bound prevents the residual update from being completely suppressed, while retaining sufficient dynamic range for spatially adaptive modulation. Regions with sufficient texture therefore receive limited compensation, whereas target boundaries and detail-deficient regions are assigned larger updates.

During the inference phase, the process is initialized from $z_{0}=H_{vis}$ and computes the fused high-frequency components via K-step Euler integration as follows:

(13) $$\;z_{k+1}\;=\;z_{k}\;+\;\frac{1}{K}v_{\theta }\left(z_{k},t_{k},H_{ir},H_{vis}\right),\;t_{k}\;=\;\frac{k}{K}$$

finally, $H_{f}\;={\;z}_{K}$ denotes the fused high-frequency component. As this update process takes place solely within the high-frequency subbands, the generative modeling is restricted strictly to edge and texture residuals, thereby preventing adverse perturbations to the low-frequency luminance and global structural configuration.

### 3.4. Low-Frequency Structural Module

**Figure 5:**
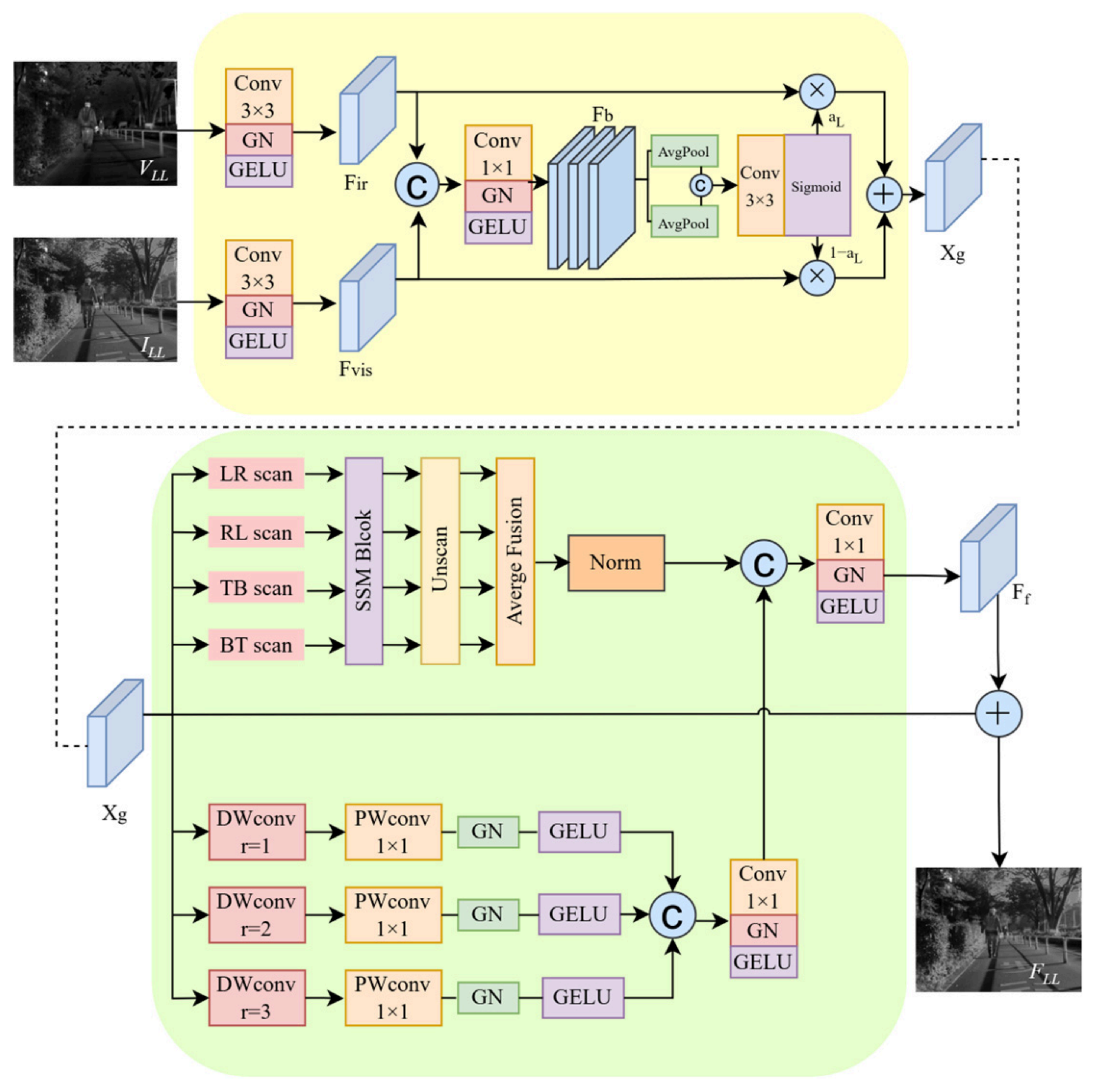
Detailed architecture of the low-frequency module.

The low-frequency branch is responsible for fusing global luminance distributions, background structures, and large-scale thermal target responses. The architecture of the low-frequency module is illustrated in Figure 5.

The low-frequency subbands determine the overall luminance distribution, background hierarchies, and contrast relationships between thermal targets and background regions within the fused image. Relying exclusively on local convolutions for low-frequency fusion inherently constrains the model with a limited receptive field, hindering its capacity to establish structural associations between distant target and background regions. Conversely, relying solely on global sequence modeling may attenuate local intensity transitions and neighborhood structural dependencies. To address these limitations, the proposed framework introduces a parallel architecture within the low-frequency branch to integrate global state-space modeling with local multi-scale convolutions, thereby endowing the low-frequency fusion process with both long-range dependency awareness and local structural rectification capabilities.

Given the infrared component $L_{ir}$ and the low-frequency visible component $L_{vis}$, the respective modality features are initially extracted via two parallel shallow convolutional branches.

(14) $$\begin{array}{c} {\;F}_{ir}=\phi_{ir}\left(L_{ir}\right),\;F_{vis}=\phi_{vis}\left(L_{vis}\right) \end{array}$$

where $\phi_{ir}\left(\cdot \right)$ and $\phi_{vis}\left(\cdot \right)$ both comprise convolutional, normalization, and non-linear activation layers to map the low-frequency subbands of individual channels into a unified feature space. Subsequently, the two feature maps are concatenated and processed by a convolutional layer 1×1 to generate the baseline low-frequency representation as follows:

(15) $$\begin{array}{c} {\;X}_{b}\;=\;\phi_{b}\left(\left[F_{ir},F_{vis}\right]\right) \end{array}$$

Building on this baseline representation, a spatial weight $\alpha_{l}$ is introduced to characterize the relative contributions of the infrared and visible low-frequency components across distinct spatial locations as follows:

(16) $$\begin{array}{c} {\;\alpha }_{L}\;=\;\sigma \left({\mathrm{Conv}}_{7\times 7}\left(\mathrm{Avg}\left(X_{b}\right),\mathrm{Max}\left(X_{b}\right)\right)\right) \end{array}$$

where $\mathrm{Avg}\left(\cdot \right)$ and $\mathrm{Max}\left(\cdot \right)$ denote channel-wise average pooling and maximum pooling operations, respectively, and σ(·) represents the sigmoid function. Instead of a simplistic global constant, this weight functions as a spatially varying low-frequency modality selection map. Specifically, regions with prominent thermal targets amplify the contribution of infrared low-frequency components, whereas regions with well-defined background structures and stable illumination predominantly preserve the visible low-frequency structural layout. The resulting guided feature is formulated as follows:

(17) $$\;X_{g}\;=\;\alpha_{L}⊙F_{ir}\;+\;\left(1\;-\;\alpha_{L}\right)⊙F_{vis}$$

To capture long-range dependencies within the low-frequency structural components, the proposed framework feeds $X_{g}$ into a multi-directional Mamba branch featuring four scanning paths. Specifically, the spatial features are flattened into sequential representations along four distinct scanning directions, namely, left-to-right, right-to-left, top-to-bottom, and bottom-to-top. These sequential representations are subsequently processed by a shared selective state-space model before being mapped back to the spatial domain. Finally, the feature maps derived from the four scanning directions are aggregated via element-wise averaging as follows:

(18) $$F_{G}\;=\;\mathrm{Norm}\left(X_{g}+\frac{1}{4}\sum_{d\in \{lr,rl,tb,bt\}}{\mathrm{Mamba}}_{d}\left(X_{g}\right)\right)$$

This configuration enables the low-frequency branch to capture long-range spatial dependencies along both horizontal and vertical axes [42,43]. While the four-directional scanning mechanism enables the model to evaluate global luminance relationships between target and background regions, the low-frequency representations still exhibit local intensity transitions and multi-scale background variations. To compensate for the limited capacity of sequential scanning to represent local neighborhood details [44,45], the proposed framework incorporates a parallel multi-dilation depthwise convolutional branch as follows:

(19) $$\;F_{L}=\phi_{l}\left(\left[{\mathrm{DWConv}}_{r-1}\left(X_{g}\right),{\mathrm{DWConv}}_{r-2}\left(X_{g}\right),{\mathrm{DWConv}}_{r-3}\left(X_{g}\right)\right]\right)$$

where r denotes the dilation rate, $\mathrm{DWConv}\left(\cdot \right)$ represents the depthwise convolution, and $\phi_{l}\left(\cdot \right)$ signifies the 1×1 convolution for channel fusion. Distinct dilation rates correspond to local receptive fields of varying sizes, enabling r=1 to focus on immediate neighborhood intensity transitions while r=2 and r=3 capture broader variations in background structures. Consequently, the multi-dilation convolutional branch and the Mamba branch are mutually complementary, since the former preserves local structural continuity while the latter models global contextual dependencies.

(20) $$\begin{array}{c} \;F_{f}=\phi_{f}\left(\left[F_{G},F_{L}\right]\right) \end{array}$$

Finally, the features from the global and local branches are concatenated and integrated via channel fusion as follows:

(21) $$\begin{array}{c} \;L_{f}\;={\;\alpha }_{L}⊙L_{ir}\;+\;\left(1\;-\;\alpha_{L}\right)⊙L_{vis}+R_{L} \end{array}$$

(22) $$\begin{array}{c} {\;R}_{L}=\rho tanh\left(\phi_{r}\left(F_{f}\right)\right) \end{array}$$

where $tanh\left(\cdot \right)$ denotes the hyperbolic tangent activation function, ${Conv}_{1\times 1}\left(\cdot \right)$ represents a 1 × 1 convolution for residual prediction, and ρ is a fixed and non-learnable coefficient that controls the amplitude of the low-frequency residual correction. Since the output of $tanh\left(\cdot \right)$ is bound within [−1, 1], the element-wise residual correction in Equation (22) is correspondingly constrained to [−ρ, ρ]. The coefficient ρ does not affect the initial infrared–visible modality weighting; instead, it regulates the extent to which the global–local residual branch refines the initially fused low-frequency representation. When ρ=0, the residual correction is disabled, and the output reduces to the initial adaptive fusion result. A larger value provides greater correction capacity for local grayscale transitions but may also cause the residual branch to modify the original luminance distribution and large-scale structure excessively.

To examine the sensitivity of the model to this coefficient and determine its final setting, a controlled parameter sweep was conducted on the MSRS dataset using ρ∈{0, 0.05, 0.10, 0.15, 0.20, 0.25, 0.30}. For each candidate value, the model was separately retrained and tested using the same fixed value of ρ, while the network architecture, dataset partition, initialization, optimization strategy, loss weights, training epochs, and evaluation procedure were kept unchanged. The overall quantitative performance improved progressively as ρ increased from 0 to 0.10 and remained close to the optimum at ρ=0.15. Further increasing ρ from 0.20 to 0.30 resulted in a gradual performance decline. These results indicate that an excessively weak residual bound limits the refinement of local grayscale transitions, whereas an overly strong correction increasingly disturbs low-frequency luminance and structural consistency. Therefore, ρ=0.10 was selected and fixed in all subsequent comparative, generalization, and ablation experiments.

### 3.5. Loss Function

Infrared and visible image fusion typically lacks a unique ground-truth reference. Consequently, directly applying a supervised reconstruction loss often causes the model to degenerate into merely fitting a single modality [46,47]. Existing unsupervised fusion methods typically regularize fused images from three aspects, namely, intensity, gradient, and structural similarity. Specifically, the intensity term preserves the infrared target response and overall luminance, the gradient term retains visible textures and source edges, while the structural term suppresses local over-sharpening or luminance drift. Building on these established conventions, the proposed framework introduces a frequency-domain divide-and-conquer strategy, partitioning the training objective into a primary image-domain constraint, a low-frequency structural constraint, and a high-frequency flow compensation constraint. The total loss function is defined as follows:

(23) $$\begin{array}{c} \;L=L_{img}+L_{lf}+L_{rf} \end{array}$$

L_img_ supervises the reconstructed fused image, L_lf_ stabilizes low-frequency luminance and structure, and L_rf_ constrains velocity-field learning and endpoint consistency in the high-frequency Rectified Flow branch. The three losses are summed without branch-level weights; all coefficients defined below are fixed hyperparameters.

The primary image-domain constraint comprises three distinct terms, namely, intensity, gradient, and structural similarity, formulated as follows:

(24) $$\begin{array}{c} \;L_{img}=\lambda_{int}{∥I_{f}-I_{t}∥}_{1}+\lambda_{g}{∥\nabla I_{f}-G_{t}∥}_{1}+\lambda_{s}\left[1-\frac{SSIM\left(I_{f},I_{ir}\right)+SSIM\left(I_{f},I_{vis}\right)}{2}\right] \end{array}$$

The three terms in Equation (24) correspond to intensity preservation, gradient transfer, and structural similarity, respectively. Their coefficients are fixed as λ_int_ = 0.6, λ_g_ = 2.0, λ_s_ = 3.0. These coefficients remain constant throughout all experiments and are excluded from the parameter set optimized by AdamW. The relatively larger weights assigned to the gradient and structural terms strengthen edge–detail transfer and structural consistency, while the intensity term maintains the basic luminance and thermal-response information of the fused image. Where the intensity objective $I_{t}$ and gradient objective $G_{t}$ are defined as follows:

(25) $$\;I_{t}=\eta \max\left(I_{ir},I_{vis}\right)+\left(1-\eta \right)\frac{I_{ir}+I_{vis}}{2}$$

(26) $$\;G_{t}=\max\left(\left|\nabla I_{ir}\right|,\left|\nabla I_{vis}\right|\right)$$

The intensity objective is formulated based on the pixel-wise maximum response and the dual-modality average response. Here, η∈[0, 1] is a fixed, non-learnable mixing coefficient that balances these two components and is set to 0.5 in all experiments. This setting preserves salient intensity responses while retaining complementary information from both source modalities, thereby alleviating overly dim target regions and limiting background luminance drift. The gradient objective employs the element-wise maximum of the gradient magnitudes extracted from both source images, enabling the fused image to preserve more pronounced edges and textures. Simultaneously, the structural similarity term evaluates the structural correlations between the fused image and both the infrared and visible modalities [48]. This term enforces overall luminance, contrast, and structural consistency, preventing the model from generating superficially sharp yet structurally unstable results through the naive amplification of high-frequency responses [49].

(27) $$\;L_{lf}=∥L_{f}\;-\;D_{L}\left(I_{t}\right){∥}_{1}$$

where $D_{L}\left(\cdot \right)$ denotes the low-frequency subband obtained via Haar wavelet decomposition. Instead of directly enhancing textures, $L_{lf}$ constrains the low-frequency output to approximate the structural profile of the intensity objective, thereby establishing a stable foundation for subsequent high-frequency compensation.

The high-frequency flow compensation constraint comprises a velocity-matching term and a high-frequency endpoint consistency term.

(28) $${\;L}_{rf}=\lambda_{flow}∥v_{\theta }\left(z_{t},t,H_{ir},H_{vis}\right)\;-\;\left(z_{1}-z_{0}\right){∥}_{2}^{2}+\lambda_{hf}∥H_{f}\;-H^{*}{∥}_{1}$$

The first term serves as the core training objective of Rectified Flow, forcing the velocity field to predict the residual trajectory from the visible high-frequency starting point $z_{0}$ to the target high-frequency endpoint $z_{1}$. The second term constrains the fused high-frequency component $H_{f}$, obtained via few-step integration, to approximate the target high-frequency component $H^{*}$. This constraint ensures that the high-frequency compensation aligns with both the local directional energy and the learnable high-frequency guidance.

The corresponding coefficients are fixed as λ_flow_ = 1.5, λ_hf_ = 3.0 in all experiments. Both coefficients are fixed during training and are not learnable parameters.

## 4. Experiments

This section begins by detailing the experimental setup and implementation details of the proposed method. To demonstrate the effectiveness of the proposed framework, comprehensive qualitative and quantitative comparisons are conducted with eleven representative methods. Furthermore, generalization experiments on alternative datasets are conducted to evaluate the robustness of the model across diverse environments. Finally, rigorous ablation studies validate the efficacy and necessity of each core component within the architecture.

### 4.1. Datasets and Experimental Setup

To comprehensively evaluate the performance of the proposed model, experiments are conducted on two publicly available datasets, namely, the MSRS and M^3^FD datasets. The MSRS dataset contains precisely registered infrared and visible image pairs encompassing daytime, nighttime, and complex road scenarios. Additionally, the M^3^FD dataset incorporates a broader range of traffic object categories and more challenging object scale variations. Specifically, 1083 image pairs from the MSRS dataset are utilized for training, while 361 image pairs are reserved for testing. To further validate the generalization capacity of the proposed method, 420 image pairs from the M^3^FD dataset are employed exclusively for evaluation.

The proposed framework is implemented in PyTorch 2.1.2 and executed on a workstation equipped with an NVIDIA GeForce RTX 4060 GPU. During training, the source images are randomly cropped into 256 × 256 pixel patches with a batch size of 2. The model is optimized via the AdamW optimizer for 100 epochs, with the initial learning rate and weight decay set to 2 × 10^−4^ and 1 × 10^−4^, respectively. For the loss configuration, the image-domain, low-frequency structural, and high-frequency Rectified Flow losses are directly summed at the branch level. Within the image-domain loss, the intensity, gradient, and structural similarity coefficients are fixed as λ_int_ = 0.6, λ_g_ = 2.0, and λ_s_ = 3.0, respectively. Within the high-frequency Rectified Flow loss, the velocity-matching and endpoint-consistency coefficients are fixed as λ_flow_ = 1.5 and λ_hf_ = 3.0, respectively. All these coefficients are predefined hyperparameters and remain unchanged during optimization. The same loss configuration is used for all comparative, generalization, and ablation experiments without dataset-specific adjustment. For the high-frequency Rectified Flow, a 5-step path sampling trajectory is adopted during training, while an 8-step Euler integration is employed by default during inference. To ensure experimental reproducibility, the random seed is set to 3407. Furthermore, all quantitative evaluations are conducted using identical test splits, uniform image sizes, and a standardized evaluation script. For color-visible images, both fusion operations and metric computations are performed exclusively on the luminance channel, thereby eliminating potential confounding effects introduced by different color restoration methods on the evaluation metrics.

### 4.2. Comparison Methods and Evaluation Metrics

To comprehensively validate the effectiveness and practicality of the proposed method, eleven representative baseline algorithms are selected for comparison, encompassing traditional transform-domain techniques, established deep learning-based approaches, and recent state-of-the-art fusion frameworks. Specifically, the competing methods include DWT [12], AGMFusion [50], ESFuse [51], DenseFuse [52], DifFusion [30], PIVFusion [17], RFFusion [32], SwinFusion [16], U2Fusion [53], PMKFuse [54], and VGDCFusion [55]. All methods are evaluated on identical test sets to compute quantitative metrics, which are subsequently analyzed alongside visual exemplars to facilitate a rigorous qualitative and quantitative assessment.

To comprehensively assess fusion performance, seven widely used metrics are selected for quantitative evaluation, namely: entropy (EN), structural similarity (SSIM), visual information fidelity (VIF), the edge preservation index (Q_abf_), spatial frequency (SF), standard deviation (SD), and peak signal-to-noise ratio (PSNR). Specifically, EN quantifies the information richness of the fused image and the degree of information preservation from the source modalities. Concurrently, SSIM, VIF, and PSNR evaluate the fused images from the perspectives of structural consistency, human visual fidelity, and reconstruction distortion, respectively. Additionally, Q_abf_ and SF characterize edge feature transfer and high-frequency texture activity, whereas SD reflects the global contrast and the dynamic range of grayscale distributions.

### 4.3. Comparative Experiments

#### 4.3.1. Qualitative Results

**Figure 6:**
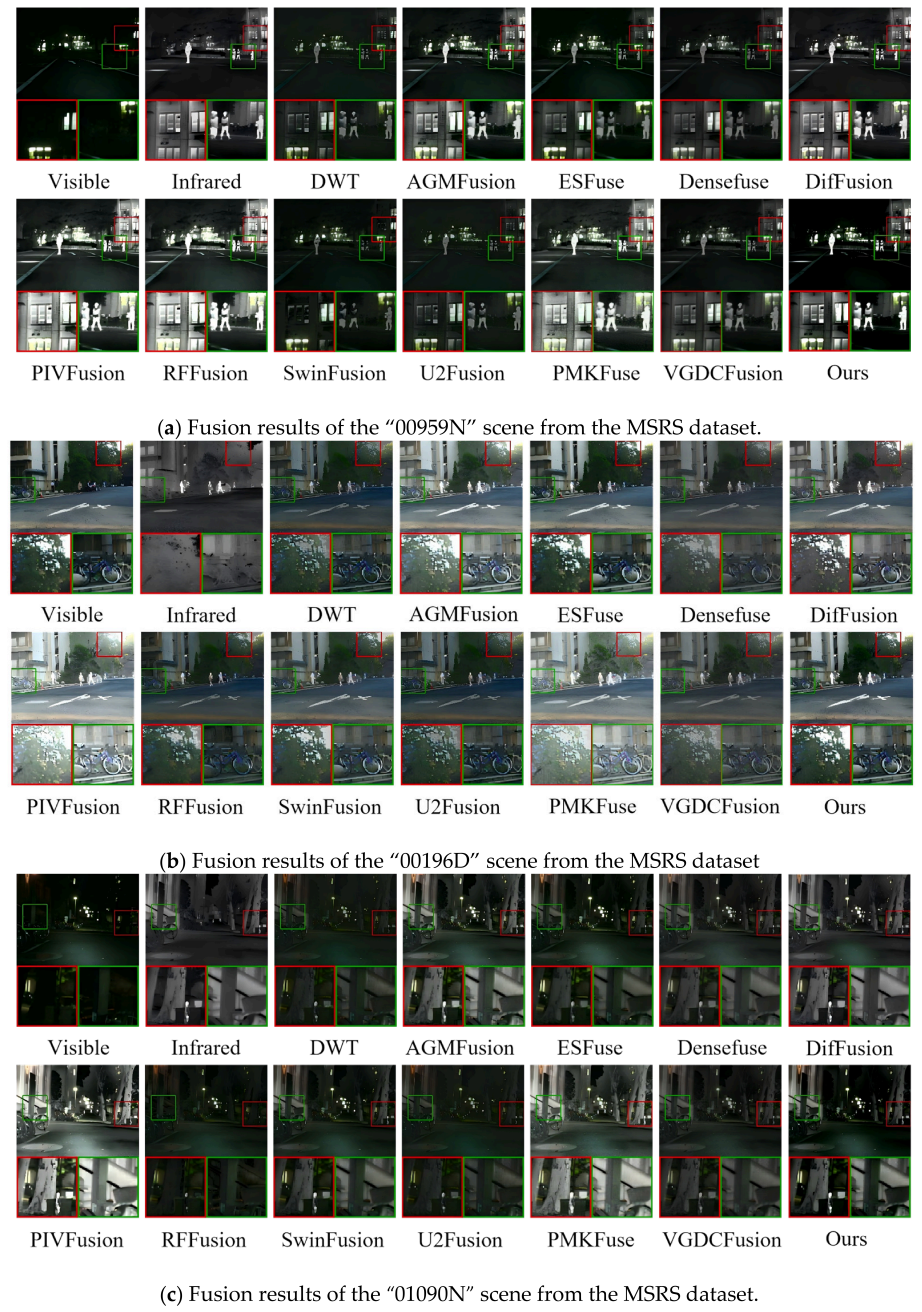
Qualitative comparison of SDC-Fusion with eleven competing methods on three representative scenes from the MSRS test set: (**a**) scene “00959N”; (**b**) scene “00196D”; and (**c**) scene “01090N”. The colored boxes indicate the regions selected for enlarged comparison.

To qualitatively evaluate the performance of different methods, three representative pairs of source images along with their fused results are selected from the MSRS dataset for visual comparison, as illustrated in Figure 6. The selected scenes encompass diverse illumination conditions, varying object scales, and distinct background complexities, thereby providing a robust benchmark to evaluate each fusion method in terms of infrared target highlighting, visible texture preservation, boundary continuity, and overall contrast.

The fusion results across the three representative scenes demonstrate that the competing methods exhibit various limitations in feature integration. Specifically, DWT, DenseFuse, and RFFusion exhibit suboptimal performance in preserving high-frequency details from the visible modality, leading to varying degrees of blurring and over-smoothing on building window panes, wall boundaries, and distant bicycle contours. Furthermore, these approaches fail to accurately delineate pedestrian leg and torso boundaries in complex nighttime scenarios. Although methods such as PIVFusion, SwinFusion, DifFusion, and PMKFuse significantly enhance local luminance and structural details, they tend to amplify background noise. This amplification introduces conspicuous graininess in shadowed regions, such as low-light alleys, and induces unnatural overexposure or harsh artifacts near intense light sources and tree canopies, thereby disrupting the inherent spatial depth hierarchy of the scene. Finally, while AGMFusion, ESFuse, and VGDCFusion effectively maintain the global scene structure, they still suffer from a minor information loss when reconstructing fine textures, such as delicate wheel spokes and pavement cracks.

In contrast, the proposed method strikes an optimal balance between low-frequency structural stability and high-frequency detail preservation. As observed from the magnified insets across various scenes, the proposed framework not only effectively reconstructs high-frequency visible textures—such as window frames, pipeline topologies, and foliage—but also precisely preserves the saliency and boundary continuity of infrared targets, such as distant pedestrians.

#### 4.3.2. Quantitative Results

Table 1 summarizes the quantitative evaluation results on the MSRS test set. To facilitate comparison, the best and second-best results are highlighted in red and blue, respectively.

As shown in Table 1, the proposed method achieves the optimal performance on five evaluation metrics, namely, SSIM, VIF, Q_abf_, SF, and PSNR, while maintaining highly competitive values for EN and SD. Compared with the best result among the eleven competing methods for each of these five metrics, SDC-Fusion improves SSIM, VIF, Q_abf_, and SF by 2.54%, 3.58%, 8.41%, and 10.44%, respectively, while increasing PSNR by 0.4071 dB. The optimal values for SSIM and PSNR demonstrate that the proposed framework exhibits a clear advantage in preserving global structural consistency and luminance stability. Furthermore, the superior VIF score indicates that the fused images align more closely with human visual perception, whereas the leading performance in Q_abf_ and SF demonstrates that the proposed method more effectively transfers edge structures and fine texture details from the source images.

Comparative analysis with the baseline methods reveals that although PIVFusion achieves the highest EN score, its corresponding SSIM and PSNR values are significantly lower. This discrepancy indicates that excessively high-frequency enhancement potentially compromises structural stability. PMKFuse achieves competitive EN, VIF, and Q_abf_ scores, but remains inferior to the proposed method in SSIM and PSNR. Additionally, the relatively lower values of Q_abf_, SF, and VIF achieved by RFFusion suggest that this algorithm fails to preserve edge textures effectively. Furthermore, while DenseFuse, U2Fusion, and VGDCFusion demonstrate stability across certain structure-based metrics, their lower SF and Q_abf_ scores indicate that pure end-to-end reconstruction frameworks are prone to detail smoothing.

#### 4.3.3. Complexity Analysis

Table 2 summarizes the model parameters, floating-point operations (FLOPs), and inference times across the eleven competing fusion methods. Runtime was measured with a batch size of 1 at an input resolution of 640 × 480. Each model was warmed up for 20 iterations and then evaluated over 100 synchronized inference runs. The reported value is the average runtime per image, excluding model loading and disk I/O but including wavelet decomposition, network inference, and inverse reconstruction. All methods were tested in FP32 on the same GPU and software environment. Compared to the majority of these approaches, the proposed model demonstrates higher efficiency in terms of both parameter size and computational complexity.

### 4.4. Generalization Experiments

#### 4.4.1. Qualitative Results

**Figure 7:**
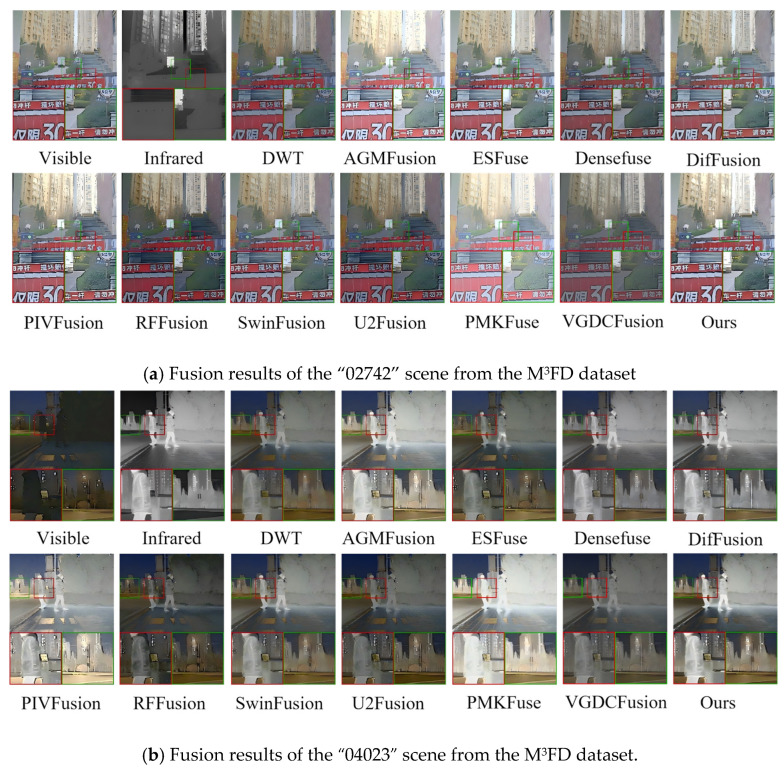
Cross-dataset qualitative comparison of SDC-Fusion with eleven competing methods on two representative scenes from the M^3^FD dataset: (**a**) scene “02742” and (**b**) scene “04023”. The colored boxes indicate the regions selected for enlarged comparison.

To evaluate the generalization capacity of the proposed model, the network trained exclusively on the MSRS dataset is directly evaluated on the M^3^FD dataset. Compared to MSRS, the M^3^FD dataset incorporates a broader range of traffic object categories, exhibits more pronounced object scale variations, and features highly complex background environments. Representative daytime and nighttime scenes are selected for visual comparison, as illustrated in Figure 7.

In complex cross-dataset scenarios, several competing methods exhibit significant generalization bottlenecks. DenseFuse, U2Fusion, and VGDCFusion exhibit varying degrees of edge blurring and over-smoothing when handling intricate structures, such as the billboard text and dense foliage highlighted by the red bounding boxes. Additionally, RFFusion, PMKFuse, and certain generative methods tend to produce excessively high overall grayscale intensities or overly dark local regions in nighttime architectural scenes or low-illumination environments, thereby severely obscuring the original facade structures and visible textures. Furthermore, methods that emphasize high-frequency enhancement, such as PIVFusion and SwinFusion, are prone to introducing substantial background noise or texture artifacts when processing dense vehicles and complex backgrounds, such as the signage and trees indicated by the green bounding boxes, thereby degrading the realism of the cross-dataset scenes.

The proposed method demonstrates robust generalization capacity. In highly complex scenarios, the framework accurately delineates and preserves billboard boundaries while maintaining a natural luminance distribution in nighttime scenes. As observed from the magnified insets within the red and green bounding boxes, the proposed approach preserves both the salient thermal responses and continuous boundaries of nighttime pedestrians without compromising background details, such as pavement patterns. Overall, the proposed method effectively suppresses artifacts and noise induced by texture over-enhancement, thereby achieving an optimal balance between highlighting infrared targets and preserving the structural integrity of the scene.

#### 4.4.2. Quantitative Results

Table 3 summarizes the quantitative evaluation results on the M^3^FD test set. To facilitate comparison, the best and second-best results are highlighted in red and blue, respectively.

As shown in Table 3, the proposed method achieves the optimal performance on five evaluation metrics, namely, SSIM, VIF, Q_abf_, SD, and PSNR, while maintaining highly competitive values for EN and SF. Compared with the best result among the eleven competing methods for each of these five metrics, SDC-Fusion improves SSIM, VIF, Q_abf_, and SD by 2.60%, 5.72%, 3.44%, and 1.32%, respectively, while increasing PSNR by 3.4118 dB. The optimal values for SSIM, PSNR, and SD demonstrate that the proposed framework exhibits a clear advantage in maintaining structural consistency and contrast stability. Additionally, the leading scores for VIF and Q_abf_ validate its capability to transfer edge textures more effectively in alignment with human visual perception.

Although PIVFusion obtains the highest EN and SF scores and PMKFuse ranks third in EN, both methods yield lower SSIM and PSNR values than SDC-Fusion. This trade-off suggests that strong high-frequency enhancement may reduce structural consistency when it is not sufficiently constrained. Additionally, while DenseFuse, U2Fusion, and VGDCFusion demonstrate stable performance across structure-oriented metrics, their lower SF and Q_abf_ scores underscore the detail-smoothing artifacts inherent in end-to-end reconstruction frameworks. Overall, the proposed method demonstrates robust generalization capacity, achieving a favorable balance among low-frequency structural stability, target-to-background contrast, and high-frequency texture details.

### 4.5. Ablation Studies

We evaluate seven configurations to isolate the contributions of the low-frequency structural module, target high-frequency guidance, gated Rectified Flow and the three loss terms. These configurations comprise six ablated variants and the complete model.

In the first variant (denoted as w/o CNN_LF), the four-directional Mamba state space model and the multi-dilation depthwise convolution in the low-frequency branch are replaced with conventional residual convolutional blocks, while keeping the remaining architecture unchanged. The second variant (denoted as w/o Guide) simplifies the learnable target high-frequency guidance module within the high-frequency branch into a fixed local maximum energy selection rule. The third variant (denoted as w/o RF) removes the gated Rectified Flow residual generation process from the high-frequency detail compensation module, directly utilizing the target high-frequency component as the final output. The fourth variant (denoted as w/o $L_{lf}$) eliminates the low-frequency structural constraint loss $L_{lf}$ from the training objective while maintaining an identical network structure and training configuration. Similarly, the fifth variant (denoted as w/o $L_{rf}$) discards the high-frequency flow compensation constraint loss $L_{rf}$ from the training objective with the remaining settings unaltered. The sixth variant (denoted as w/o $L_{img}$) removes the image-domain loss $L_{img}$ from the training objective while retaining the remaining settings. Finally, the complete framework (denoted as All) retains all components and constraint losses to evaluate the baseline fusion performance.

**Figure 8:**
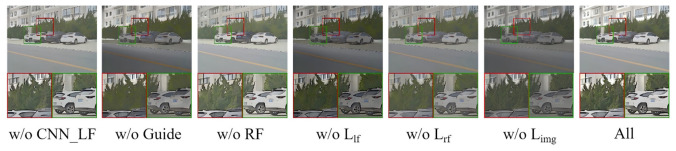
Qualitative ablation comparison of six model variants and the complete SDC-Fusion model on a representative scene from the MSRS test set. All variants are trained and evaluated under identical settings, with only the indicated module or loss term removed.

Figure 8 illustrates the fusion results obtained under seven distinct experimental configurations. As indicated by the magnified regions, clear visual discrepancies exist among the generated images. Specifically, the six ablation variants exhibit varying degrees of edge blurring, texture smoothing, and degraded target-to-background contrast. In contrast, the proposed SDC-Fusion method consistently outperforms these alternative configurations in terms of detail preservation, edge sharpness, target-to-background contrast, and color fidelity.

Table 4 summarizes the quantitative evaluation metrics for the fused images generated by the competing models on the MSRS dataset.

Quantitative results demonstrate that the metrics for the six ablated variants exhibit varying degrees of degradation compared to the complete model. In contrast, SDC-Fusion achieves the optimal performance across all metrics. Specifically, removing the low-frequency module (w/o CNN_LF) and simplifying the guidance mechanism (w/o Guide) lead to a marked decline in structural metrics (SSIM and PSNR) and edge-based metrics (Q_abf_ and SF), respectively. Eliminating the gated Rectified Flow component (w/o RF) causes a substantial decrease in texture information (EN and SF), confirming the efficacy of gated Rectified Flow in preserving detailed textures. Furthermore, removing either the constraint loss $L_{lf}$ or $L_{rf}$ severely compromises the performance. In particular, in the absence of $L_{rf}$, SSIM and VIF drop to their lowest values, indicating that velocity field matching is indispensable for suppressing synthesis noise and ensuring structural fidelity. Removing $L_{img}\;$also leads to performance degradation, with SSIM, VIF, and PSNR decreasing to 0.6187, 0.6764, and 60.5839, respectively, confirming the effectiveness of the image-domain loss in maintaining the overall fusion quality.

## 5. Conclusions

Under the dual constraints of low-frequency macrostructures and high-frequency micro-edge details, the proposed SDC-Fusion method significantly enhances the quality of fused images in terms of edges, textures, and contrast. Specifically, the high-frequency branch incorporates a gated detail enhancement module based on Rectified Flow, thereby substantially enriching the fine textures of the fused images. Simultaneously, the low-frequency branch employs a specialized module that integrates adaptive low-frequency weights, a four-directional Mamba global scan, and local multi-dilated depthwise convolutions. This design facilitates a natural transition of local grayscale values while preserving the underlying background structure. By leveraging the complementary advantages of both branches, SDC-Fusion advances the performance of infrared and visible image fusion, yielding a substantial increase in detail richness while ensuring overall structural stability.

Although SDC-Fusion achieves substantial advancements in image fusion quality, room for improvement remains regarding its computational efficiency and adaptability across physical hardware platforms and complex, dynamic environments. Future work will investigate structured pruning and mixed-precision or INT8 quantization to reduce computational and storage overhead while preserving the low-frequency structural information and high-frequency details emphasized by the proposed framework. In addition, deployment-oriented optimization will focus on the four-directional Mamba scanning process and the few-step Euler integration in the gated Rectified Flow branch, with particular attention to the trade-off between fusion quality and inference efficiency. Task-driven constraints and robustness under complex real-world conditions will also be further explored to improve the practical applicability of SDC-Fusion.

## Figures and Tables

**Table 1:** Quantitative comparison of eleven competing methods and SDC-Fusion on the MSRS test set.

Method	EN	SSIM	VIF	Q_abf_	SF	SD	PSNR
DWT	5.0492	0.6013	0.4889	0.3405	7.2195	20.3359	55.5645
AGMFusion	6.5583	0.691	0.8605	0.5741	11.7124	44.5032	61.9821
ESFuse	6.5672	0.7012	0.8487	0.5962	10.8008	45.3817	61.9726
DenseFuse	6.1872	**0.7234**	0.7594	0.4821	9.6573	28.1378	62.6104
DifFusion	6.5605	0.684	0.8273	0.5828	11.6305	41.9016	63.1723
PIVFusion	**7.0433**	0.5602	0.8614	0.4529	12.2283	34.9194	58.989
RFFusion	5.8124	0.7199	0.6168	0.3158	9.9537	**49.4295**	**66.7225**
SwinFusion	6.1035	0.5261	0.8556	0.5977	**12.586**	24.5781	64.0125
U2Fusion	6.0432	0.6803	0.8289	0.5705	8.2275	38.3312	64.5678
PMKFuse	6.5486	0.6847	**0.8628**	**0.6126**	12.0734	43.8127	63.5849
VGDCFusion	6.1482	0.7075	0.8036	0.6084	9.1276	31.9462	62.4385
Ours	**6.6436**	**0.7418**	**0.8937**	**0.6641**	**13.9002**	**46.5141**	**67.1296**

The results are averaged over 361 infrared–visible image pairs. The best and second-best results are highlighted in red and blue, respectively.

**Table 2:** Comparison of model complexity and inference efficiency among eleven competing methods and the proposed SDC-Fusion.

Method	Parameters (M)	FLOPs (G)	Inference Time (s)
DWT	0	0.0195	0.0561
AGMFusion	0.014	4.311	0.0315
ESFuse	4.648	202.367	1.3124
DenseFuse	0.074	54.151	1.0968
DifFusion	14.296	189.98	15.3106
PIVFusion	0.573	218.74	3.1153
RFFusion	65.573	1244.91	4.3184
SwinFusion	0.927	298.738	2.2007
U2Fusion	0.659	405.174	0.9215
PMKFuse	0.047	3.8678	0.3141
VGDCFusion	53.154	940.98	7.231
Ours	0.535	67.5	0.1545

Parameters are reported in millions (M), FLOPs in billions (G), and inference time in seconds (s).

**Table 3:** Cross-dataset quantitative comparison of eleven competing methods and SDC-Fusion on the M^3^FD dataset.

Method	EN	SSIM	VIF	Q_abf_	SF	SD	PSNR
DWT	6.529	0.7014	0.5394	0.4698	12.2804	27.7228	62.3722
AGMFusion	6.7693	0.6498	0.7531	0.5284	14.5440	37.4083	58.3773
ESFuse	6.7161	0.6818	**0.7881**	**0.6224**	14.2220	36.8635	60.4193
DenseFuse	6.6627	0.7027	0.6822	0.5339	10.6356	32.9772	61.8764
DifFusion	6.6907	0.6697	0.4709	0.3839	11.9803	33.7072	60.9726
PIVFusion	**7.2723**	0.6232	0.6901	0.5051	**15.7062**	**41.9004**	59.3452
RFFusion	6.6245	0.7027	0.629	0.4683	11.5583	34.926	63.1023
SwinFusion	6.8788	0.6688	0.77	0.57087	15.2489	41.5671	60.1706
U2Fusion	6.5512	**0.7182**	0.6153	0.5449	11.0546	28.7989	**63.4441**
PMKFuse	7.0084	0.6417	0.7316	0.5638	14.8215	40.0863	60.2841
VGDCFusion	6.3173	0.7084	0.6748	0.6007	10.2846	33.7185	62.4814
Ours	**7.0139**	**0.7369**	**0.8332**	**0.6438**	**15.4326**	**42.4524**	**66.8559**

The results are averaged over 420 infrared–visible image pairs. The best and second-best results are highlighted in red and blue, respectively.

**Table 4:** Quantitative results of six ablation variants and the complete SDC-Fusion model on the MSRS test set.

Method	EN	SSIM	VIF	Q_abf_	SF	SD	PSNR
w/oCNN_LF	6.3507	0.6697	0.7453	0.5899	12.0103	38.7572	61.4226
w/oGuide	6.3611	0.6611	0.7831	0.5744	13.7070	41.8231	62.8763
w/oRF	6.4532	0.6582	0.7083	0.4969	11.5546	34.8189	62.5641
w/oL_lf_	6.4411	0.6414	0.6494	0.5213	12.1704	35.8128	59.3222
w/oL_rf_	6.4793	0.5688	0.5731	0.5464	12.3440	38.4683	59.3773
w/oL_img_	6.4132	0.6187	0.6764	0.5668	12.7421	39.2846	60.5839
All	**6.6436**	**0.7418**	**0.8937**	**0.6641**	**13.9002**	**46.5141**	**67.1296**

All variants are trained and evaluated under identical settings, with only the indicated module or loss term removed. The best results are highlighted in red.

## Data Availability

The original data presented in the study are openly available in MSRS Dataset at https://github.com/Linfeng-Tang/MSRS (accessed on 3 March 2026).

## References

[1] Zhao W., Wang W., Wang H., He Y., Lu H. (2026). CDTFusion: Crossing domain and task for infrared and visible image fusion. IEEE Trans. Pattern Anal. Mach. Intell..

[2] Fu D., Li Z., Fan W., Wang Q. (2026). USF-Net: Infrared-visible image fusion via unified semantics and context modulation. Sensors.

[3] Zhou W., Wu W., Zhou H. Semantic-aware infrared and visible image fusion. Proceedings of the 2021 4th International Conference on Robotics, Control and Automation Engineering (RCAE).

[4] Li X., Chen R., Wang J., Chen W., Zhou H., Ma J. (2025). CASPFuse: An infrared and visible image fusion method based on dual-cycle crosswise awareness and global structure-tensor preservation. IEEE Trans. Instrum. Meas..

[5] Liu X., Lin Y., Zhang S., Wang X., Shi Y., Lin L. (2025). AngularFuse: A closer look at angle-based perception for spatial-sensitive multi-modality image fusion. arXiv.

[6] Sun T., Xiang D., Ding T., Fang X., Qi Y., Zhao Z. (2025). Modality-aware infrared and visible image fusion with target-aware supervision. arXiv.

[7] Jiang M., Wang Z., Kong J., Zhuang D. (2024). MCFusion: Infrared and visible image fusion based on multiscale receptive field and cross-modal enhanced attention mechanism. J. Electron. Imaging.

[8] Li X., Duan Z., Chang J. (2024). Multi-feature decomposition and transformer-fusion: An infrared and visible image fusion network based on multi-feature decomposition and transformer. J. Electron. Imaging.

[9] Wang X., Xi J., Yang F., Yang Y., Li M. (2026). PFI-Net: A parallel feature interaction network for infrared and visible target detection. Pattern Recognit..

[10] Li H., Xu T., Wu X.-J., Lu J., Kittler J. (2023). LRRNet: A novel representation learning guided fusion network for infrared and visible images. IEEE Trans. Pattern Anal. Mach. Intell..

[11] Zhao W., Xie S., Zhao F., He Y., Lu H. MetaFusion: Infrared and visible image fusion via meta-feature embedding from object detection. Proceedings of the IEEE/CVF Conference on Computer Vision and Pattern Recognition (CVPR).

[12] Li H., Manjunath B.S., Mitra S.K. (1995). Multisensor image fusion using the wavelet transform. Graph. Models Image Process..

[13] Zou Y., Wu J., Ye B., Cao H., Feng J., Wan Z., Yin S. (2025). Infrared and visible image fusion based on multi-scale transform and sparse low-rank representation. Front. Phys..

[14] Yang Y., Gao C., Ming Z., Guo J., Leopold E., Cheng J., Zuo J., Zhu M. (2023). LatLRR-CNN: An infrared and visible image fusion method combining latent low-rank representation and CNN. Multimed. Tools Appl..

[15] Wang Z., Chen Y., Shao W., Li H., Zhang L. (2022). SwinFuse: A residual Swin Transformer fusion network for infrared and visible images. IEEE Trans. Instrum. Meas..

[16] Ma J., Tang L., Fan F., Huang J., Mei X., Ma Y. (2022). SwinFusion: Cross-domain long-range learning for general image fusion via Swin Transformer. IEEE/CAA J. Autom. Sin..

[17] Hua D., Chen Q., Wu Z., Zuo Y., Wen W., Fang Y. (2025). Perceptual transform fusion of infrared and visible images. IEEE Trans. Circuits Syst. Video Technol..

[18] Liu X., Mersha B.W., Hirota K., Dai Y. (2025). Infrared and visible image fusion with overlapped window Transformer. J. Adv. Comput. Intell. Intell. Inform..

[19] Wu X., Cao Z.-H., Huang T.-Z., Deng L.-J., Chanussot J., Vivone G. (2025). Fully-connected Transformer for multi-source image fusion. IEEE Trans. Pattern Anal. Mach. Intell..

[20] Li H., Wu X.-J. (2024). CrossFuse: A novel cross attention mechanism based infrared and visible image fusion approach. Inf. Fusion.

[21] Zhao Z., Bai H., Zhang J., Zhang Y., Xu S., Lin Z., Timofte R., Van Gool L. CDDFuse: Correlation-driven dual-branch feature decomposition for multi-modality image fusion. Proceedings of the IEEE/CVF Conference on Computer Vision and Pattern Recognition (CVPR).

[22] Li Q., Yan B., Luo D. (2024). Infrared and visible image fusion method based on hierarchical attention mechanism. J. Electron. Imaging.

[23] Li Y., Shi Y., Pu X., Zhang S. (2024). Infrared and visible image fusion based on global context network. J. Electron. Imaging.

[24] Peng S., Zhu X., Deng H., Deng L.-J., Lei Z. (2024). FusionMamba: Efficient remote sensing image fusion with state space model. IEEE Trans. Geosci. Remote Sens..

[25] Li Z., Pan H., Zhang K., Wang Y., Yu F. (2024). MambaDFuse: A Mamba-based dual-phase model for multi-modality image fusion. arXiv.

[26] Hu Q., Peng Y., U K.T., Zhao S. (2025). Infrared and visible image fusion using a state-space adversarial model with cross-modal dependency learning. Mathematics.

[27] Liu J., Fan X., Huang Z., Wu G., Liu R., Zhong W., Luo Z. Target-aware dual adversarial learning and a multi-scenario multi-modality benchmark to fuse infrared and visible for object detection. Proceedings of the IEEE/CVF Conference on Computer Vision and Pattern Recognition (CVPR).

[28] Ma J., Yu W., Liang P., Li C., Jiang J. (2019). FusionGAN: A generative adversarial network for infrared and visible image fusion. Inf. Fusion.

[29] Ho J., Jain A., Abbeel P. (2020). Denoising diffusion probabilistic models. Adv. Neural Inf. Process. Syst..

[30] Yue J., Fang L., Xia S., Deng Y., Ma J. (2023). Dif-Fusion: Toward high color fidelity in infrared and visible image fusion with diffusion models. IEEE Trans. Image Process..

[31] Zhao Z., Bai H., Zhu Y., Zhang J., Xu S., Zhang Y., Zhang K., Meng D., Timofte R., Van Gool L. DDFM: Denoising diffusion model for multi-modality image fusion. Proceedings of the IEEE/CVF International Conference on Computer Vision (ICCV).

[32] Wang Z., Zhang J., Guan T., Zhou Y., Li X., Dong M., Liu J. (2025). Efficient rectified flow for image fusion. Adv. Neural Inf. Process. Syst..

[33] Wang L., Wang X. (2025). Infrared and visible image fusion based on cross-modality feature alignment. Proc. SPIE.

[34] Zhao W., Cui H., Wang H., He Y., Lu H. (2025). FreeFusion: Infrared and visible image fusion via cross reconstruction learning. IEEE Trans. Pattern Anal. Mach. Intell..

[35] Sang H., Peng X. (2025). Nighttime pedestrian detection using crossed wavelet fusion networks. J. Electron. Imaging.

[36] Dharejo F.A., Zhou Y., Deeba F., Jatoi M.A., Khan M.A., Mallah G.A., Ghaffar A., Chhattal M., Du Y., Wang X. (2021). A deep hybrid neural network for single image dehazing via wavelet transform. Optik.

[37] Dong G., Ma Y., Basu A. (2021). Feature-guided CNN for denoising images from portable ultrasound devices. IEEE Access.

[38] Liu X., Gong C., Liu Q. Flow straight and fast: Learning to generate and transfer data with rectified flow. Proceedings of the Eleventh International Conference on Learning Representations (ICLR).

[39] Liu X., Zhang X., Ma J., Peng J., Liu Q. InstaFlow: One step is enough for high-quality diffusion-based text-to-image generation. Proceedings of the Twelfth International Conference on Learning Representations (ICLR).

[40] Lipman Y., Chen R.T.Q., Ben-Hamu H., Nickel M., Le M. Flow matching for generative modeling. Proceedings of the Eleventh International Conference on Learning Representations (ICLR).

[41] Feng T., He K., Yue G., Yin J. (2025). LIGFusion: A light-integrated and guided fusion method for multimodal image fusion. J. Electron. Imaging.

[42] Zhu L., Liao B., Zhang Q., Wang X., Liu W., Wang X. (2024). Vision Mamba: Efficient visual representation learning with bidirectional state space model. Proc. Mach. Learn. Res..

[43] Liu Y., Tian Y., Zhao Y., Yu H., Xie L., Wang Y., Ye Q., Jiao J., Liu Y. (2024). VMamba: Visual state space model. Adv. Neural Inf. Process. Syst..

[44] Wang Y., Wang G., Chen C., Pan Z. (2019). Multi-scale dilated convolution of convolutional neural network for image denoising. Multimed. Tools Appl..

[45] He S., Liu J., Yang Z., Yuan R. (2026). A multi-scale fusion and attention-guided infrared small target detection network. Digit. Signal Process..

[46] Wang Z., Simoncelli E.P., Bovik A.C. Multiscale structural similarity for image quality assessment. Proceedings of the 37th Asilomar Conference on Signals, Systems and Computers.

[47] Liu Y., Qi Z., Cheng J., Chen X. (2024). Rethinking the effectiveness of objective evaluation metrics in multi-focus image fusion: A statistic-based approach. IEEE Trans. Pattern Anal. Mach. Intell..

[48] Johnson J., Alahi A., Fei-Fei L., Leibe B., Matas J., Sebe N., Welling M. (2016). Perceptual losses for real-time style transfer and super-resolution. Computer Vision—ECCV 2016.

[49] Cai R., Zhou F., Chen W., Wang H., Ge Y., Xie Q., Chen Y. (2026). A two-stage framework for infrared image enhancement under long-range turbulence. Opt. Commun..

[50] Liu S., Lan X., Chen W., Zhang Z., Qiu C. (2024). AGMFusion: A real-time end-to-end infrared and visible image fusion network based on adaptive guidance module. IEEE Sens. J..

[51] Liu W., Tan H., Cheng X., Li X. (2024). ESFuse: Weak edge structure perception network for infrared and visible image fusion. Electronics.

[52] Li H., Wu X.-J. (2019). DenseFuse: A fusion approach to infrared and visible images. IEEE Trans. Image Process..

[53] Xu H., Ma J., Jiang J., Guo X., Ling H. (2022). U2Fusion: A unified unsupervised image fusion network. IEEE Trans. Pattern Anal. Mach. Intell..

[54] Sun Y., Dong M., Zhu L. (2025). Rethinking the approach to lightweight multi-branch heterogeneous image fusion frameworks: Infrared and visible image fusion via the parallel Mamba-KAN framework. Opt. Laser Technol..

[55] Zhao J., Zhang T., Cui G. (2026). A VLM guided network coupling degradation modeling for degradation aware infrared and visible image fusion. Sci. Rep..

